# Supplementary material for: Predicting Pancreatic Ductal Adenocarcinoma Occurrence Up to 10 Years in Advance Using Features of the Main Pancreatic Duct in Pre-Diagnostic CT Scans
Source: Cancers (Basel). 2025 Jun 4;17(11):1886. doi: 10.3390/cancers17111886 (PMC12153928; doi:10.3390/cancers17111886)
Supplement: Supplementary file 1 [file cancers-17-01886-s001.zip › Supplementary Figures.pdf]

Supplementary Figures

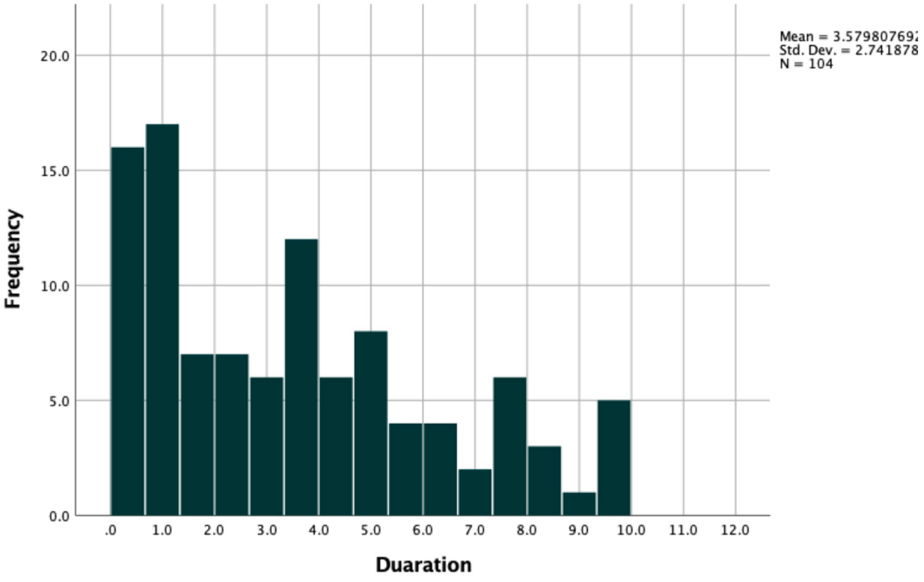

Figure S1. The distribution of time interval between pre-diagnostic CT and PDAC occurrence. Fifty-one cases (49.0%) developed PDAC within 6 months to 3 years, 32 cases (30.8%) developed PDAC within 3-6 years and 21 cases (20.2%) developed to PDAC within 6-10 years.

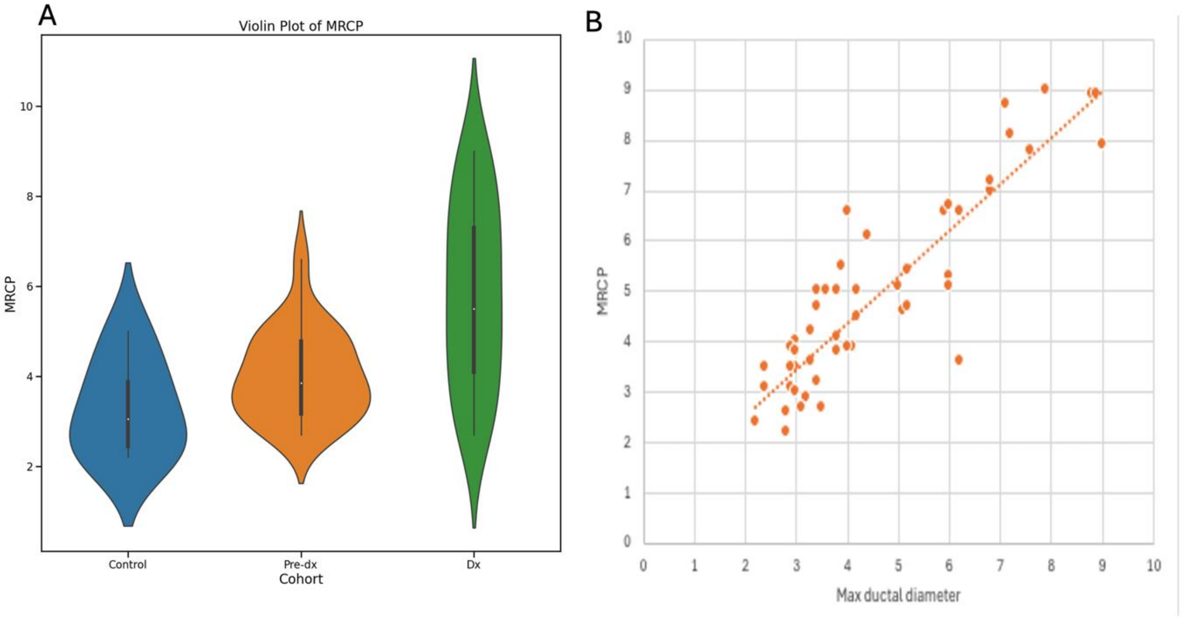

Figure S2 The violin plots of the trend of MPD Diameter among across control, pre-diagnostic and diagnostic cohorts measured on MRCP (A), and the correlation of Max ductal Diameter on CT with diameter on MRCP (B). The Pearson correlation coefficient between two modalities is 0.894. (Pre-dx: pre-diagnostic cohort; Dx: diagnostic cohort)

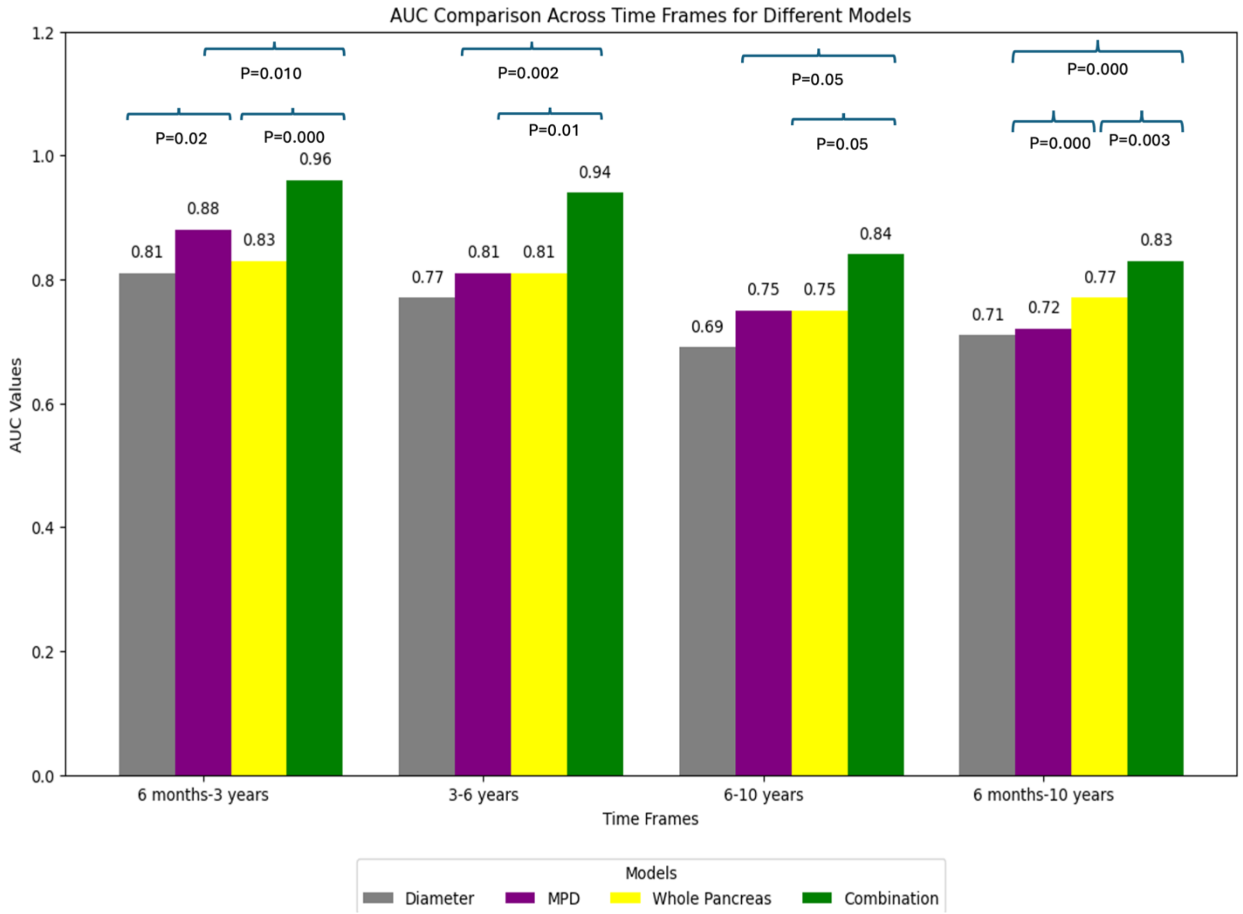

Figure S3 AUC values across different time frames for four models. The bar plot illustrates the Area Under the Curve (AUC) values for the model from diameter and volume(grey), MPD including radiomics, diameter and volume (purple), Pancreas (yellow), combination of both MPD and pancreas (green). The time intervals between pre-diagnostic date to diagnostic date are 6 months-3 years, 3-6 years, 6-10 years and 6 months-10 years. The AUC values and significant P values are displayed upper in the bar.

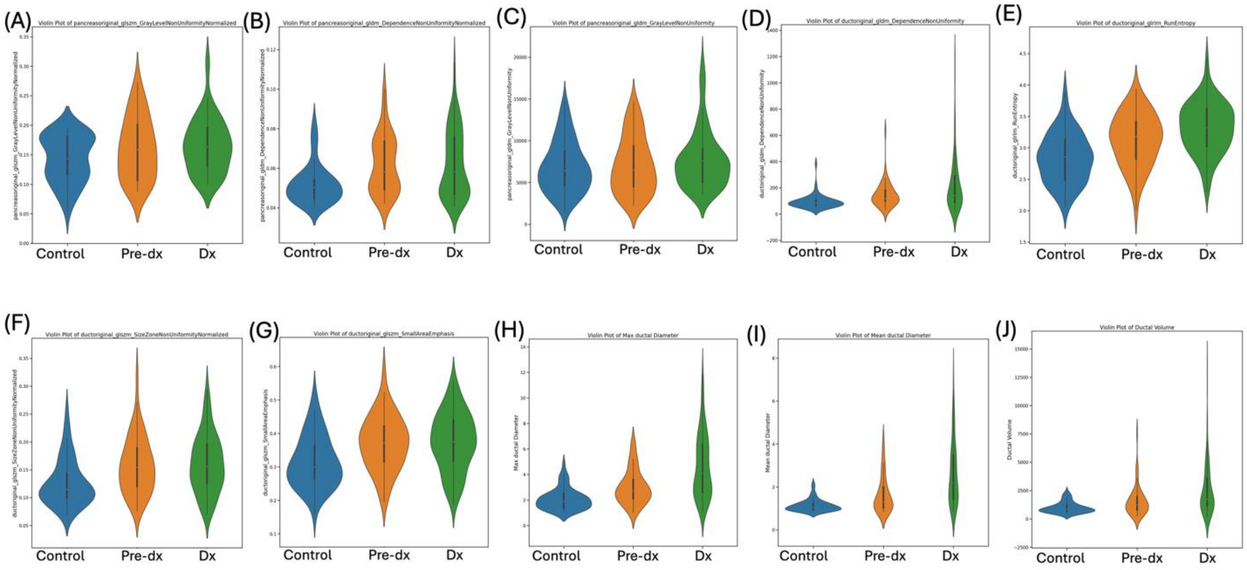

Figure S4 Violin plots illustrating the distribution and density of selected radiomic features from the pancreas (pancreasoriginal_glszm_GrayLevelNonUniformityNormalized (A), pancreasoriginal_gldm_DependenceNonUniformityNormalized (B), pancreasoriginal_gldm_GrayLevelNonUniformity (C)) and the main pancreatic duct (MPD) (ductoriginal_gldm_DependenceNonUniformity (D), ductoriginal_glrlm_RunEntropy (E), ductoriginal_glszm_SizeZoneNonUniformityNormalized (F), ductoriginal_glszm_SmallAreaEmphasis (G), Max ductal Diameter (H), Mean ductal Diameter (I), Ductal Volume (J)) across control (blue), pre-diagnostic (orange), and diagnostic cohorts in predicting PDAC occurrence within 6 months-3 years. Each violin plot displays the distribution and density of the radiomic features within each cohort. Statistical analysis reveals significant differences ($P < 0.05$) between the control and pre-diagnostic cohorts. (Pre-dx: pre-diagnostic cohort; Dx: diagnostic cohort)

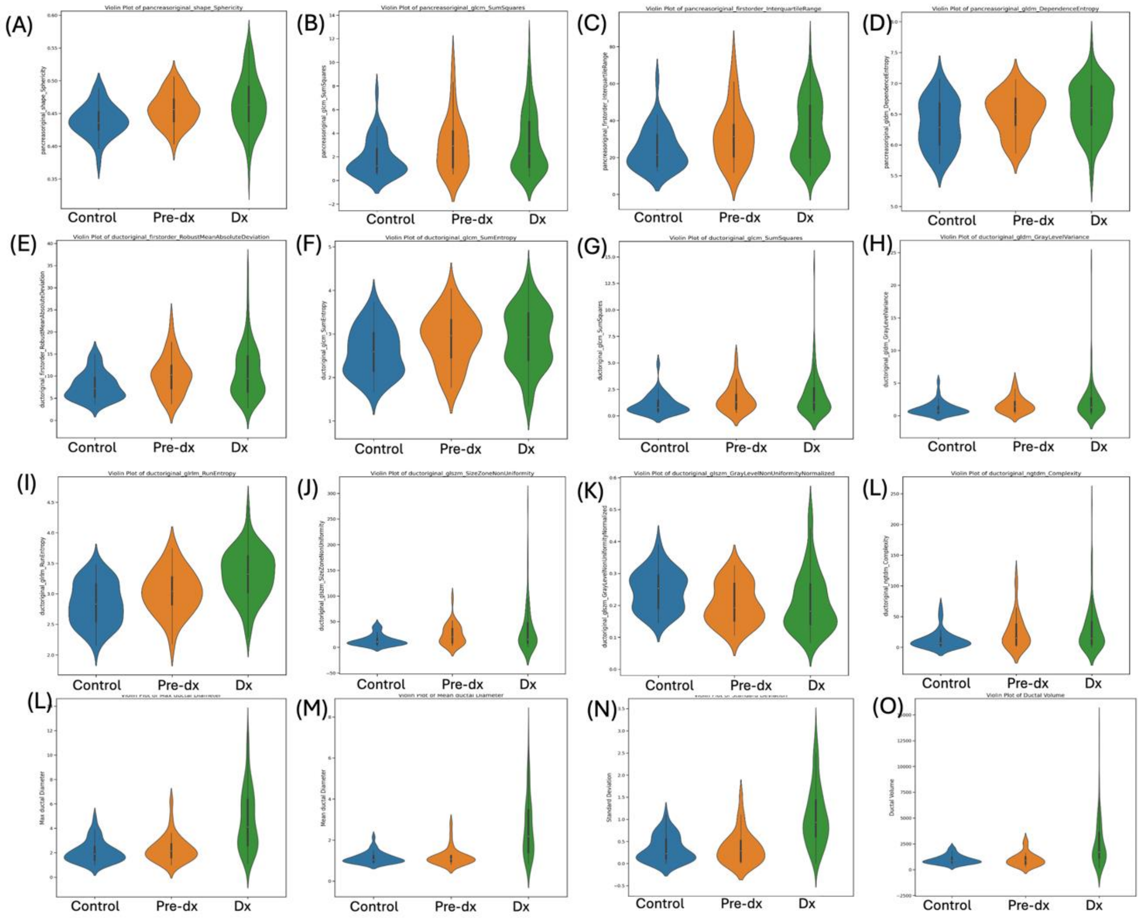

Figure S5 Violin plots illustrating the distribution and density of selected radiomic features from the pancreas (pancreasoriginal_shape_Sphericity(A), pancreasoriginal_glcm_SumSquares (B), pancreasoriginal_firstorder_InterquartileRange (C), pancreasoriginal_gldm_DependenceEntropy (D)) and the main pancreatic duct (MPD) (ductoriginal_firstorder_RobustMeanAbsoluteDeviation (E), ductoriginal_glcm_SumEntropy (F), ductoriginal_glcm_SumSquares (G), ductoriginal_gldm_GrayLevelVariance (H), ductoriginal_glrlm_RunEntropy (I), ductoriginal_glszm_SizeZoneNonUniformity (J) , ductoriginal_glszm_GrayLevelNonUniformityNormalized (K), ductoriginal_ngtdm_Complexity (L), Max ductal Diameter (M), Mean ductal Diameter (N), Standard Deviation, Ductal Volume (O)) across control (blue), pre-diagnostic (orange), and diagnostic cohorts in predicting PDAC occurrence within 3-6 years. Each violin plot displays the distribution and density of the radiomic features within each cohort. Statistical analysis reveals significant differences ($P < 0.05$) between the control and pre-diagnostic cohorts.

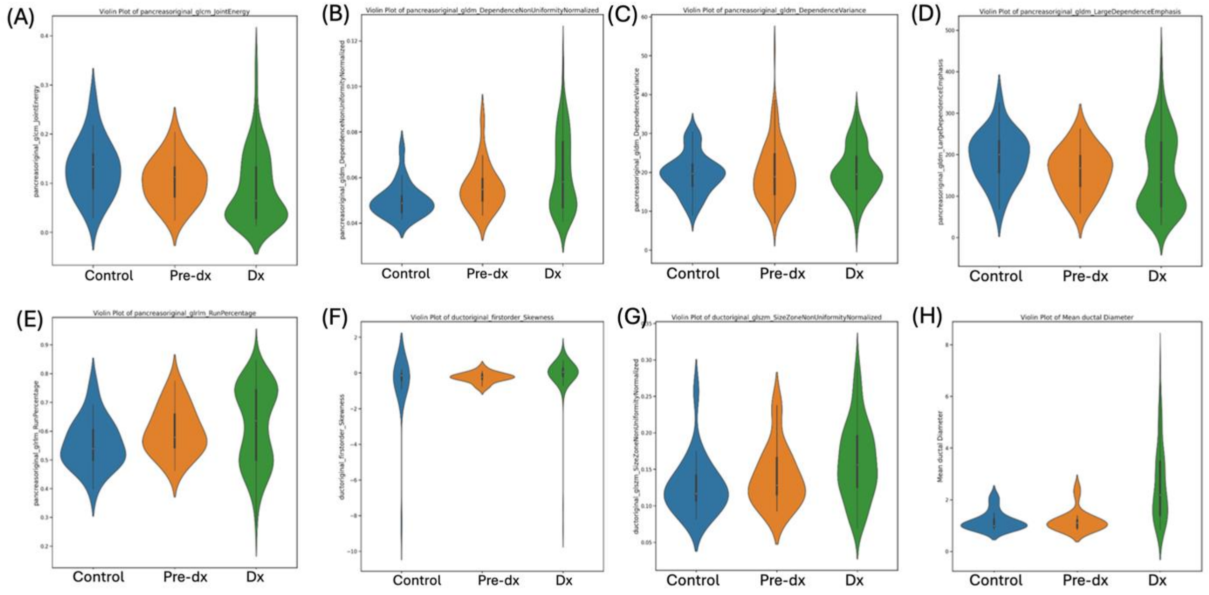

Figure S6 Violin plots illustrating the distribution and density of selected radiomic features pancreasoriginal_glcm_JointEnergy (A), pancreasoriginal_gldm_DependenceNonUniformityNormalized (B), pancreasoriginal_gldm_DependenceVariance (C), pancreasoriginal_gldm_LargeDependenceEmphasis (D), pancreasoriginal_glrlm_RunPercentage (E), ductoriginal_firstorder_Skewness (F), ductoriginal_glszm_SizeZoneNonUniformityNormalized (G), Mean ductal Diameter (H)) across control (blue), pre-diagnostic (orange), and diagnostic cohorts in predicting PDAC occurrence within 6-10 years. Each violin plot displays the distribution and density of the radiomic features within each cohort. Statistical analysis reveals significant differences ($P < 0.05$) between the control and pre-diagnostic cohorts. (Pre-dx: pre-diagnostic cohort; Dx: diagnostic cohort)

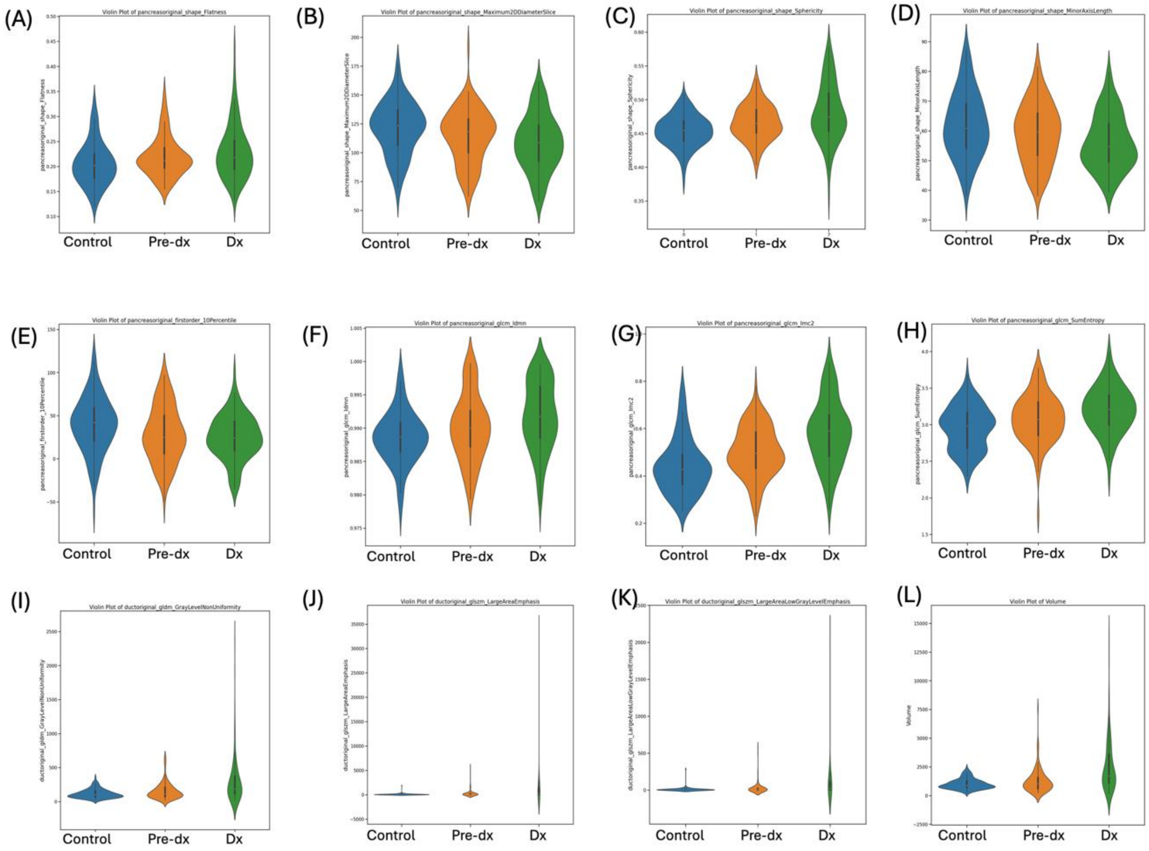

Figure S7 Violin plots illustrating the distribution and density of selected radiomic features from the pancreas (pancreasoriginal_shape_Flatness (A), pancreasoriginal_shape_Maximum2DDiameterSlice (B), pancreasoriginal_shape_Sphericity ©, pancreasoriginal_shape_MinorAxisLength (D), pancreasoriginal_firstorder_10Percentile (E), pancreasoriginal_glcm_Idmn (F), pancreasoriginal_glcm_Imc2 (G), pancreasoriginal_glcm_SumEntropy (H)) and the main pancreatic duct (MPD) (ductoriginal_gldm_GrayLevelNonUniformity (I), ductoriginal_glszm_LargeAreaEmphasis (J), ductoriginal_glszm_LargeAreaLowGrayLevelEmphasis (K), Mean ductal Diameter (L)) across control (blue), pre-diagnostic (orange), and diagnostic cohorts in predicting PDAC occurrence for overall cases (6 months-10 years). Each violin plot displays the distribution and density of the radiomic features within each cohort. Statistical analysis reveals significant differences ($P < 0.05$) between the control and pre-diagnostic cohorts. . (Pre-dx: pre-diagnostic cohort; Dx: diagnostic cohort)

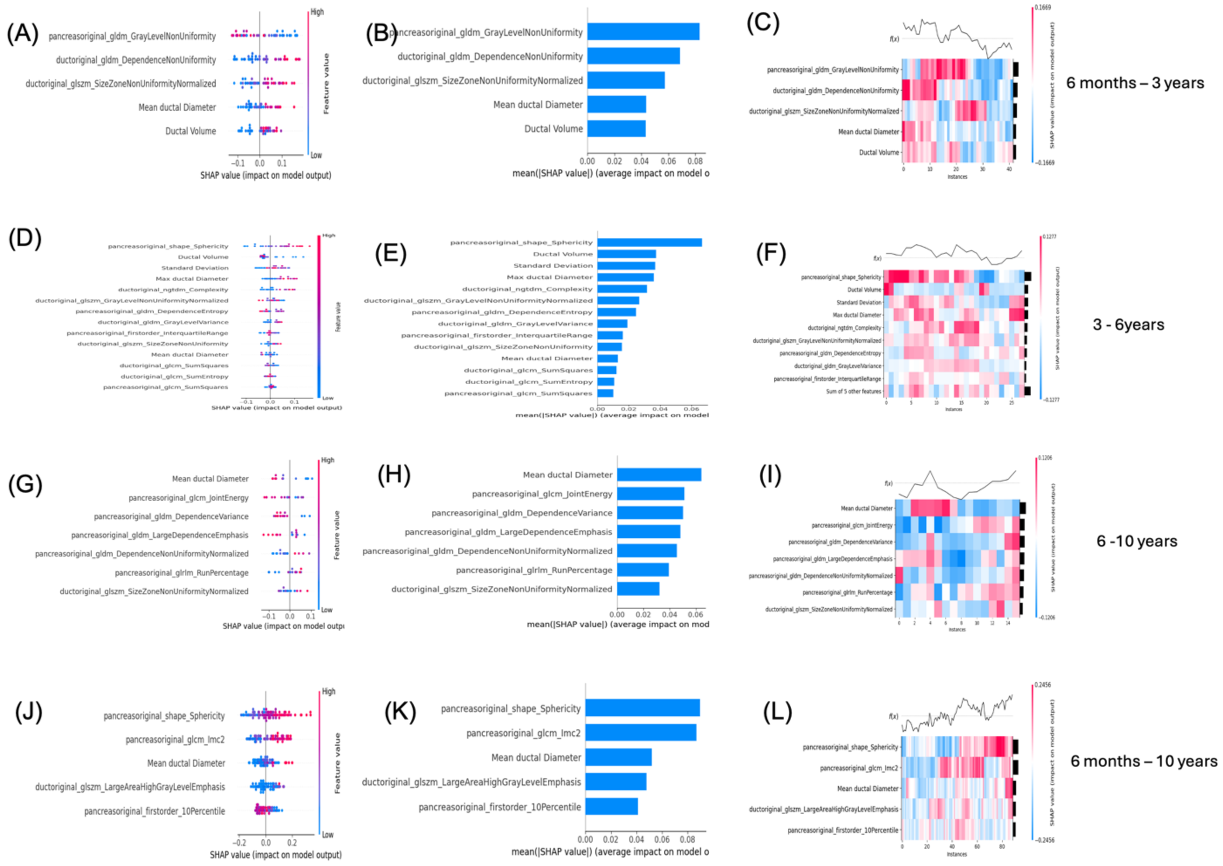

Figure S8 Global model explanation by the SHAP method of different timeframe(A,B,C within 6 months-3 years in advance; D,E,F within 3-6 years in advance, G,H,I within 6-10years in advance; J,K,L within 6 months-10 years in advance) when combined the MPD features and radiomic features of pancreas. The SHAP beeswarm plots for the selected features in the models of different time frame (A,D,G,J) . Each dot represents a patient for each feature, with red denoting a higher feature value and blue denoting a lower feature value. The x-axis represents the SHAP values that describe the impact of each feature on model prediction. Positive SHAP values indicate an increased risk of PDAC, whereas negative SHAP values indicate a decreased risk. The dots are stacked vertically to show density. The SHAP bar charts for the selected features in the models of different time frame (B,E,H,K) show **the contribution of each feature** to a model's predictions. The SHAP heatmap plots in the models of different time frame (C,F,I,L) shows the direction and intensity of influence for each feature of all cases in the model. (SHAP, Shapley Additive explanation; PDAC, pancreatic ductal adenocarcinoma; MPD, main pancreatic duct)
