# Supplementary material for: Predicting Pancreatic Ductal Adenocarcinoma Occurrence Up to 10 Years in Advance Using Features of the Main Pancreatic Duct in Pre-Diagnostic CT Scans
Source: Cancers (Basel). 2025 Jun 4;17(11):1886. doi: 10.3390/cancers17111886 (PMC12153928; doi:10.3390/cancers17111886)
Supplement: Supplementary file 1 [file cancers-17-01886-s001.zip › Supplementary Tables.pdf]

Supplementary Tables

Table S1 The scan protocol and parameters of CT and MRCP

CT parameters

	Manufacters	Detector Rows	Slice Thickness (mm)	kVp	mAs	Field of View (FOV)	Reconstruction Algorithm	Acquisition Matrix	Contrast Phase	Preprocessing Steps
CT	GE LightSpeed VCT	64-slice	5	120	Auto (typically 260 mAs)	40cm	ASIR	512×512	Portal venous phase (70–80 sec post-contrast injection)	DICOM to NIfTI conversion, isotropic resampling (1 mm)
CT	Siemens SOMATOM Force	Dual-source	2.5	120	250 mAs (auto exposure control)	40cm	Br36	512×512	Portal venous phase (65-75 sec)	DICOM conversion, co-registration for serial scans
CT	Philips Brilliance 64	64-slice	3.0	120	Auto	40cm	Filtered Back Projection	512×512	Portal venous phase (65-75 sec)	DICOM to NIfTI conversion, isotropic resampling to 1 mm, image normalization
CT	TOSHIBA Aquilion ONE	320-slice CT scanner	5	120	250 mAs (auto exposure control)	40cm	AIDR	512×512	Portal venous phase (65-75 sec)	DICOM to NIfTI conversion, isotropic resampling to 1 mm, image normalization

MR parameters

	Manufacters	Field Strength	Slice Thickness (mm)	Gap	TR	TE	FA	Acquisition Matrix	Sequence	Preprocessing Steps
MRCP	Siemens 3.0T Prisma, SymphonyTim	3.0T	3.0	0.5	4739	501	140	384×371	3D Cor_T2_spc_rst	DICOM to NIfTI conversion,

										denoising filter applied
MRCP	GE 3.0T Signa HDxt	3.0	3	0.5-1	2,000–2,500	100-120	90	320 × 320	Navigator-triggered 3D MRCP	Motion correction, isotropic interpolation

Table S2 Selected features and coefficients

Time frames		Selected features	coefficients
6 months -3 years	Diameter	Ductal Volume	1.453
		Max ductal Diameter	2.518
		Mean ductal Diameter	4.619
		Standard Deviation	-2.970
	MPD (Diameter+Radiomics)	Max ductal Diameter	0.673
		Mean ductal Diameter	1.326
		ductoriginal_gldm_DependenceNonUniformity	1.002
		ductoriginal_glrlm_RunEntropy	0.685
		ductoriginal_glszm_SizeZoneNonUniformityNormalized	1.539
		ductoriginal_glszm_SmallAreaEmphasis	1.351
	Pancreas	pancreasoriginal_gldm_DependenceNonUniformityNormalized	2.447
		pancreasoriginal_gldm_GrayLevelNonUniformity	-0.912
		pancreasoriginal_glszm_GrayLevelNonUniformityNormalized	-1.134
	MPD+pancreas	Ductal Volume	0.196
		Mean ductal Diameter	2.212
		ductoriginal_gldm_DependenceNonUniformity	0.132
		ductoriginal_glszm_SizeZoneNonUniformityNormalized	1.399
		pancreasoriginal_gldm_GrayLevelNonUniformity	-0.906
3-6 years	Diameter	Ductal Volume	-0.606
		Max ductal Diameter	1.526
		Mean ductal Diameter	0.767
		Standard Deviation	-1.293
	MPD (Diameter+Radiomics)	Max ductal Diameter	-0.083
		ductoriginal_firstorder_RobustMeanAbsoluteDeviation	0.958
		ductoriginal_glszm_GrayLevelNonUniformityNormalized	-1.087
		ductoriginal_glszm_SizeZoneNonUniformity	1.274
		ductoriginal_ngtdm_Complexity	0.687
	Pancreas	pancreasoriginal_glcm_SumSquares	0.616
		pancreasoriginal_shape_Sphericity	0.859
	MPD+pancreas	Ductal Volume	0.479
		Max ductal Diameter	1.620
		Mean ductal Diameter	0.638
		Standard Deviation	-2.258
		ductoriginal_glcm_SumEntropy	-1.574
		ductoriginal_glcm_SumSquares	0.405

		ductoriginal_gldm_GrayLevelVariance	-1.159
		ductoriginal_glszm_GrayLevelNonUniformityNormalized	-1.925
		ductoriginal_glszm_SizeZoneNonUniformity	2.244
		ductoriginal_ngtdm_Complexity	0.972
		pancreasoriginal_firstorder_InterquartileRange	-0.175
		pancreasoriginal_glcm_SumSquares	1.153
		pancreasoriginal_gldm_DependenceEntropy	-0.813
		pancreasoriginal_shape_Sphericity	2.670
6-10 years	Diameter	Ductal Volume	-0.361
		Max ductal Diameter	-0.608
		Mean ductal Diameter	0.689
		Standard Deviation	0.624
	MPD (Diameter+Radiomics)	ductoriginal_firstorder_Skewness	0.753
		ductoriginal_glszm_SizeZoneNonUniformityNormalized	1.253
		Mean ductal Diameter	1.146
	Pancreas	pancreasoriginal_gldm_DependenceNonUniformityNormalized	-0.203
		pancreasoriginal_gldm_DependenceVariance	-1.578
		pancreasoriginal_gldm_LargeDependenceEmphasis	0.677
	MPD+pancreas	Mean ductal Diameter	1.071
		ductoriginal_glszm_SizeZoneNonUniformityNormalized	1.191
		pancreasoriginal_glcm_JointEnergy	-0.394
		pancreasoriginal_gldm_DependenceNonUniformityNormalized	0.666
		pancreasoriginal_gldm_DependenceVariance	-1.022
		pancreasoriginal_gldm_LargeDependenceEmphasis	-0.702
		pancreasoriginal_glrlm_RunPercentage	0.496
6 months -10 years	Diameter	Ductal Volume	-0.679
		Max ductal Diameter	0.761
		Mean ductal Diameter	2.234
		Min ductal Diameter	-0.060
		Standard Deviation	-1.129
	MPD (Diameter+Radiomics)	ductoriginal_glszm_LargeAreaEmphasis	0.760
		ductoriginal_glszm_LargeAreaLowGrayLevelEmphasis	0.270
		ductoriginal_gldm_GrayLevelNonUniformity	0.260
	Pancreas	pancreasoriginal_firstorder_10Percentile	-0.819
		pancreasoriginal_glcm_Idmn	0.499
		pancreasoriginal_glcm_Imc2	0.297
		pancreasoriginal_glcm_SumEntropy	0
		pancreasoriginal_shape_Flatness	0.149
		pancreasoriginal_shape_Maximum2DDiameterSlice	-0.299
		pancreasoriginal_shape_MinorAxisLength	-0.315
		pancreasoriginal_shape_Sphericity	0.369
	MPD+pancreas	Mean ductal Diameter	0.7728
		ductoriginal_glszm_LargeAreaHighGrayLevelEmphasis	-0.1560
		pancreasoriginal_firstorder_10Percentile	-0.1989
		pancreasoriginal_glcm_Imc2	0.7076
		pancreasoriginal_shape_Sphericity	0.5879

Table S3 lists the selected features with the definition and potential relevance[45-48]

Time frame		Selected radiomic features	Definition	Potential Relevance
6 months-3 years	Derived from MPD	original_glszm_SizeZoneNonUniformityNormalized	Measure the variability of size zone volumes within the image, where lower value indicates more homogeneity among the zone size.	Identifying the variance in sizing of zones within the ductal construction, that could be critical to an abnormality analysis
		original_glszm_SmallAreaEmphasis	Assess the distribution of small size zones, with a higher value indicating a great number of smaller zones and fine textures.	Indicates the presence of finer textures in the duct. Higher values suggest a higher concentration of smaller zones.
		original_gldm_DependenceNonUniformity	Evaluate the similarity of dependence across the image, with lower value reflecting more homogeneity among dependencies.	Higher values suggest more heterogeneity in the dependency of gray levels in the duct.
		original_glrlm_RunEntropy	Measures the uncertainty or randomness in the distribution of run lengths and gray levels.	A higher value indicates more heterogeneity within the duct.
	Derived from the whole pancreas	original_gldm_DependenceNonUniformityNormalized	Assess the similarity of dependence throughout the image, with lower value signifying more homogeneity in dependencies	Identifying the diversity in zone sizes within the pancreas, which could be relevant for detecting abnormalities. a lower value indicating more homogeneity among dependencies in the image.
		original_gldm_GrayLevelNonUniformity	Measures the similarity of gray-level intensity values in the image with lower GLN value corresponding to greater similarity in intensity values.	High value indicates that there is a high variation in pixel intensities within the region of interest. Lower values indicate that the gray levels are more uniformly distributed.
		original_glszm_GrayLevelNonUniformityNormalized	Quantifies the variability of gray-level intensity values in the image, normalized by the number of zones.	Lower values indicating a greater similarity in intensity values, suggest more uniform textures.
3-6 years	Derived from MPD	original__firstorder_RobustMeanAbsoluteDeviation	Calculates the mean distance of all intensity values from the Mean Value based on the subset of image with gray levels between the 10th and 90th percentile. The Robust Mean Absolute Deviation (RMAD) is similar to standard deviation but is less sensitive to outliers due to its focus on the median.	High value might indicate significant variability within the tissue, which could be relevant in differentiating between normal and abnormal regions.
		original_glcm_SumEntropy	Represents the sum of intensity differences between neighboring pixels.	Higher value could be indicative of tissue

			heterogeneity and might correlate with abnormal ductal changes, such as fibrosis or early neoplastic processes.
	original_glcm_SumSquares	Measure the variance in neigboring intensity level pairs around the mean intensity level in the GLCM.	A higher value indicates greater variability.
	original_gldm_GrayLevelVariance	Evaluates the variance of gray level intensities within the zones.	Variability in the gray levels could indicate changes in tissue density, reflect tissue heterogeneity.
	original_glszm_SizeZoneNonUniformity	Assesses the variability of size zone volumes in the image, where lower value suggests greater homogeneity in zone sizes.	Assessing the complexity and heterogeneity of the pancreatic duct A high value indicates that there is a high variability in the sizes of these zones, meaning there are both small and large zones present with different gray levels.
	original_glszm_GrayLevelNonUniformityNormalized	Measure the variability of gray-level intensity values in the image, normalized for size.	Suggest irregularities in tissue architecture. Correspond to structural abnormalities, such as dilated or irregular ducts, potentially indicating the presence of neoplastic changes.
	original_ngtdm_Complexity	An image is considered complex when it has numerous primitive components, such as. rapid changes in gray level intensity, indicating non-uniformity. These metric measures local variation in gray levels across the image and reflect show much each pixel differs from the average of its neighbors.	High complexity could suggest a more disordered structure, which may be indicative of disease.
Derived from the whole pancreas	original_shape_Sphericity	Measure the roundness of tumor region relative to a sphere. It is a dimensionless measure, independent of scale and orientation, with values range from 0-1 where 1 indicates a perfect sphere which has the smallest possible surface area for a given volume).	Assess the regularity and smoothness of the pancreatic structure.to a sphere.
	original_glcm_SumSquares	Sum of Squares, or variance represents the distribution of neighboring intensity level pairs around the mean intensity level in the GLCM.	High sum squares may reflect more complex and varied tissue structures, which could help in identifying areas of interest, such as tumors.
	original_firstorder_InterquartileRange	Measure the intensity values between the 25th and 75th percentiles within a region.	A higher value indicates greater heterogeneity in tissue density.

		original_gldm_DependenceEntropy	Quantify the complexity of the spatial dependencies between pixels, reflecting the randomness of relationships among neighboring pixels.	Higher value might indicate complex tissue structures and disorganized tissue patterns.
6-10 years	Derived from MPD	original_firstorder_Skewness	Measures the asymmetry in the distribution of values around the mean Which can be positive or negative depending on the direction of the tail and concentration of distribution. It also measures the minimum intensity value in the duct region of interest (ROI).	Represent the asymmetry in the distribution of intensities within the pancreatic duct.
		original_glszm_SizeZoneNonUniformityNormalized	Assess the variability of size zone volumes throughout the image, where lower value indicates more homogeneity among the zone sizes.	Identifying the diversity in zone sizes within the ductal structure, which could be relevant for detecting abnormalities.
		original_glcm_JointEnergy	Measure homogeneous patterns in the image.	Lower value might signify more disorganized tissue, potentially indicate structural abnormalities in the pancreas due to tumor growth or inflammation .
		original_gldm_LargeDependenceEmphasis	Measures the distribution of large dependencies, where higher value indicates larger dependence and more homogeneous textures.	**High value indicates** that the image has larger, uniform areas with consistent intensity values. This could mean that the pancreas has regions of homogeneity.
		original_gldm_DependenceVariance	Evaluate the variance in the size of dependence size within the image.	Assessing the complexity and heterogeneity of textures within the pancreatic tissue. Higher values indicate more variability in dependencies.
		original_gldm_DependenceNonUniformityNormalized	Measures the variability in the size of homogeneous zones in the pancreas, normalized by the total number of zones.	Identifying the diversity in zone sizes within the pancreas, which could be relevant for detecting abnormalities.
		original_glrlm_RunPercentage	Measure texture coarseness by taking the ratio of number of runs to the number of voxels within the region of interest (ROI).	Higher value could correlate with disrupted or irregular pancreatic tissue architecture.
6 months-10 years	Derived from MPD	original_glszm_LargeAreaEmphasis	Quantifies the distribution of large area size zones, where value reflect the presence of larger size zones and coarser textures in the duct.	Might correlate with tissue homogeneity.
		original_glszm_LargeAreaLowGrayLevelEmphasis	Measure the proportion of joint distribution of larger size zones with lower gray-level values within the image.	May indicate fibrosis surrounding the ductal structure. The breakdown in the architecture, ductal dilation and

			scarring manifest as larger, low-intensity regions on imaging.
	original_gldm_GrayLevelNonUniformity	Assesses the similarity of gray-level intensity values in the image, with lower value indicating greater similarity in intensity values.	Reflect heterogeneity in tissue texture, possibly indicating irregularities in pancreatic duct structure caused by early tumor development or inflammation.
Derived from the whole pancreas	original_firstorder_10Percentile	Represents the 10th percentile of voxel intensities in the region of interest (ROI), indicating the lower range of intensity values.	Identifying hypoattenuating areas within the pancreas.
	original_glcm_Idmn	Measures the local homogeneity of the image.	Higher homogeneity in texture might indicate benign conditions
	original_glcm_Imc2	Assesses the correlation between the probability distributions of intensity level i and j, reflecting the texture complexity.	Reflect complexity within the pancreas. PDAC potentially increasing the texture complexity.
	original_glcm_SumEntropy	Represents the sum of intensity differences between neighboring pixels.	Higher values can indicate more complex or disordered tissue architecture.
	original_shape_Flatness	Measures the relationship between the largest and smallest principal components of the shape in the region of interest (ROI).	A value closer to 1 indicates a flatter or disk-like shape.
	original_shape_Maximum2DDiameterSlice	Defined as the largest pairwise Euclidean distance between tumor surface mesh vertices in the row-column (usually axial) plane.	The largest diameter of the ROI in the 2D slice. Lower value could reflect chronic changes or atrophy.
	original_shape_MinorAxisLength	Yield the second-largest axis length of the ROI-enclosing ellipsoid, calculated using the largest principal component $\lambda minor$.	Lower value could indicate pancreatic deformation related to PDAC or atrophy related to chronic pancreatitis.
	original_shape_Sphericity	Measure the roundness of the shape of the specific region relative to a sphere, with values between 0 and 1, where 1 represents a perfect sphere which has. has the smallest surface area for a given volume.	Assess the regularity and smoothness of the pancreatic structure to a sphere.
